# 

**Published:** 1909-08-07

**Authors:** 


### BOOKS RECEIVED.

H. J. Glaisher.

"Lectures on Hysteria." By Thomas D. Savill, M.D.

John Murray.

"Children in Health and Disease." By David Forsyth, M.D., D.Sc.

"In a Good Cause," Stories and Verses on Behalf of the Hospital for Sick Children. By various authors.

J. B. Lippincott Co.

"International Clinics." 19th Series. Vol. II.

J. Wright and Co., Bristol.

"Arthritis Deformans." By R. Llewellyn Jones, M.B.

John Wright and Sons, Ltd.

"Pye's Surgical Handicraft." Fifth edition. By W. H. Clayton-Greene, B.A., M.B., etc.

### THE GRADUATES' MEDICAL SCHOOLS.

DIARY FOR THE WEEK, AUGUST 9 to 14.

**THE POST-GRADUATE COLLEGE, West London Hospital, Hammersmith, W.**

**At 10 a.m.**
**August 9 and 12,** Surgical Registrar, **Demonstration.**

**At 12 noon.**
**August 9,** Dr. Bernstein, **Pathological Demonstration.**

**At 3 p.m.**
**August 9,** Mr. Bidwell, **Surgical Cases.**
**August 10,** Dr. Pritchard, **Medical Cases.**

**At 5 p.m.**
**August 11,** Mr. Armour, **Clinical Lecture.**
**August 12,** Mr. Keetley, **Clinical Lecture.**
**August 13,** Dr. Davis, **Tonsillitis.**

The Secretary of the Seamen's Hospital Society reports that the summer session of the London School of Tropical Medicine at this hospital has just drawn to a close and that it has been one of the most successful on record. Fifty-one students have taken out the course. The accommodation at the school provides for only forty students in the general laboratory and about five in each of the special departments. With the present classes the school has been much over-crowded. Thanks to the generosity of a friend of the school, the laboratories will be enlarged during the long vacation, and it is hoped that when the school reopens in October there will be sufficient accommodation for all who desire to go through the curriculum, even if they are more numerous than in the present session.

THE HOSPITAL August 7, 1909.

*Name* ..........................................

*Address* ..........................................

..........................................

This Coupon must accompany manuscript or contributions intended for The Hospital.

